# Altered circular RNA expression profiles in the non-ischemic thalamus in focal cortical infarction mice

**DOI:** 10.18632/aging.103424

**Published:** 2020-07-08

**Authors:** Fangming Li, Chuqiao Li, Xiaoqiang Li, Yudi Li, Yulan Zhong, Li Ling

**Affiliations:** 1 Department of Neurology, Shenzhen University General Hospital, Shenzhen University Clinical Medical Academy, Shenzhen, Guangdong, China; 2 Department of Neurology, Nanfang Hospital, Southern Medical University, Guangzhou, Guangdong, China; 3 Department of Neurology, Affiliated Xiaolan Hospital, Southern Medical University (Xiaolan People’s Hospital), Zhongshan, Guangdong, China; 4 Department of Neurology, Shenzhen Hospital, Southern Medical University, Shenzhen, Guangdong, China

**Keywords:** focal cortical infarction, thalamus, circular RNA, high-throughput sequencing

## Abstract

Focal cerebral infarction leads to secondary changes in non-ischemic areas remote from but connected to the infarct site. Circular RNAs (circRNAs) are involved in the pathophysiological processes of many diseases. However, the expression and roles of circRNAs in non-ischemic remote regions after ischemic stroke remain unknown. In this study, adult male C57BL/6J mice were subjected to permanent distal middle cerebral artery occlusion (MCAO) to establish focal cortical infarction. High-throughput sequencing was used to profile the circRNA expression in the mouse ipsilateral thalamus at 7 and 14 d after MCAO. Bioinformatics analyses were conducted to predict the function of the differential expressed circRNAs’ host and target genes. Compared with sham group, a total of 2659 circRNAs were significantly altered in the ipsilateral thalamus at 7 or 14 d after MCAO in mice. Among them, 73 circRNAs were significantly altered at both two time points after stroke. GO and KEGG analyses indicated that circRNAs plays important roles in secondary thalamic neurodegeneration and remodeling after focal cortical infarction. This is the first study to profile the circRNA expression in non-ischemic region of ischemic stroke, suggesting that circRNAs may be therapeutic targets for reducing post-stroke secondary remote neurodegeneration.

## INTRODUCTION

Stroke is one of the leading causes of death and severe long-term disability in the world. Ischemic stroke is the most common type of stroke. Although many efforts have been made to protect ischemic penumbra, it is challenging to develop effective clinical strategies due to the limited therapeutic time windows after stroke onset. In the last decades, increasing studies have focused on post-stroke secondary neurodegeneration in non-ischemic remote regions which have synaptic connections with the ischemic lesion [1–5]. Neuronal loss, microglial activation, gliosis, and axonal degeneration are confirmed as the main pathologic changes in non-ischemic remote areas after focal cerebral infarction. Notably, secondary remote neurodegeneration has been shown to be closely related to long-term neurological outcomes of patients with ischemic stroke [6, 7]. Our previous study identified new blood vessels and neuronal cells in the ipsilateral thalamus of adult rats with focal cortical infarction, suggesting that self-repair occurred in non-ischemic remote areas after ischemic stroke [8]. Since the occurrence of secondary remote neurodegeneration in non-ischemic brain areas is relatively late and progresses slowly, it is feasible to develop therapeutic strategies to reduce secondary remote injuries in patients with ischemic stroke. Therefore, it is essential to explore the molecular mechanisms underlying secondary remote neurodegeneration and remodeling after focal cerebral infarction.

Circular RNA (circRNA) is a novel class of non-coding RNAs with covalently closed-loop structure, which is highly stable and conservative. Most Circular RNAs (circRNAs) can act as microRNAs (miRNAs) sponges to regulate endogenous gene expressions through the competing RNA network. CircRNAs have been detected in specific spatial and temporal patterns in the brain [9]. Interaction analysis of circRNAs and disease-associated miRNAs suggest that circRNAs play critical roles in many diseases [10–15]. Notably, the expression of circRNA was significantly altered in the ischemic brain regions of mice [16, 17]. However, the expression profiles and potential roles of circRNA in the non-ischemic remote regions after ischemic stroke remain unknown.

In this study, we investigated the dynamical expressions of circRNA in the non-ischemic ipsilateral thalamus in adult mice with focal cortical infarction. The bioinformatics analyses were used to predict the potential functions of significantly altered circRNAs’ genes in the ipsilateral thalamus. Three circRNAs were selected from those significantly altered in the ipsilateral thalamus at both two time points (7 and 14 d after stroke) for further validation via quantitative reverse transcription-PCR (qRT-PCR). Our results suggested that circRNAs may play key roles in the pathophysiological processes of secondary thalamic neurodegeneration and remodeling after focal cortical infarction.

## RESULTS

### The secondary ipsilateral thalamus damage in the permanent distal middle cerebral artery occlusion (MCAO) mice

**Figure 1:**
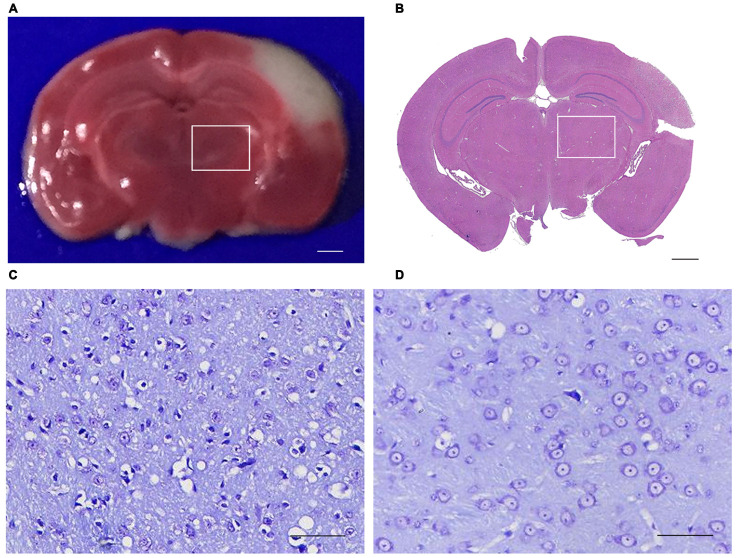
**Triphenyltetrazolium chloride (TTC), Hematoxylin and eosin (H&E) and Nissl Staining of the brain section.** (**A**) TTC staining showed the red region is normal brain tissue and the white region is infarction (bar=100μm). (**B**) H&E staining showed that the primary infarction is confined to the left cortex and does not involve the ipsilateral thalamus in mice (bar=100μm). (**C**, **D**) Nissling staining of the ipsilateral thalumus of the focal cortical infarction mice and sham mice (bar=50μm). (The rectangle indicates the non-ischemic ipsilateral thalamus).

As triphenyltetrazolium chloride (TTC) and Hematoxylin and Eosin (H&E) staining shown, permanent distal MCAO resulted in an ipsilateral infarction, which was confined to the ipsilateral cerebral cortex but without involvement of the ipsilateral thalamus (Figure 1A, 1B). Thus, this ipsilateral thalamus region is a non-ischemic brain subregion, which remotes from the infarct lesion. No infarction was detected in the sham group mice.

Secondary thalamic neurodegeneration after local cortical infarction was characterized by Nissl Staining. At 14 d after operation, obvious cytoplasmic and nuclear shrinkage were observed in the ipsilateral thalamic neurons of MCAO mice. Compared to the sham group, the number of intact neurons in the ipsilateral thalamus decreased significantly in the MCAO group (Figure 1C, 1D). These results suggested that the secondary thalamic neurodegeneration is not directly caused by distal MCAO, but secondary to the primary cortical infarction.

### Altered circRNA expression profiles in the ipsilateral thalamus after focal cortical infarction

High-throughput sequencing was used to profile circRNA expression in the mouse ipsilateral thalamus at 7 and 14 d after MCAO in this study. A total of 37548 circRNAs were identified in all the ipsilateral thalamus samples. Compared with the sham group, 2659 circRNAs were differentially expressed (|log2FoldChange| > 1.0 and *p* < 0.05) in the non-ischemic ipsilateral thalamus at 7 or 14 days after MCAO. Among them, 73 circRNAs were significantly altered at both two time points (7 and 14 d after MCAO). No differences in the distribution of circRNA expression patterns were found in all samples.

**Figure 2:**
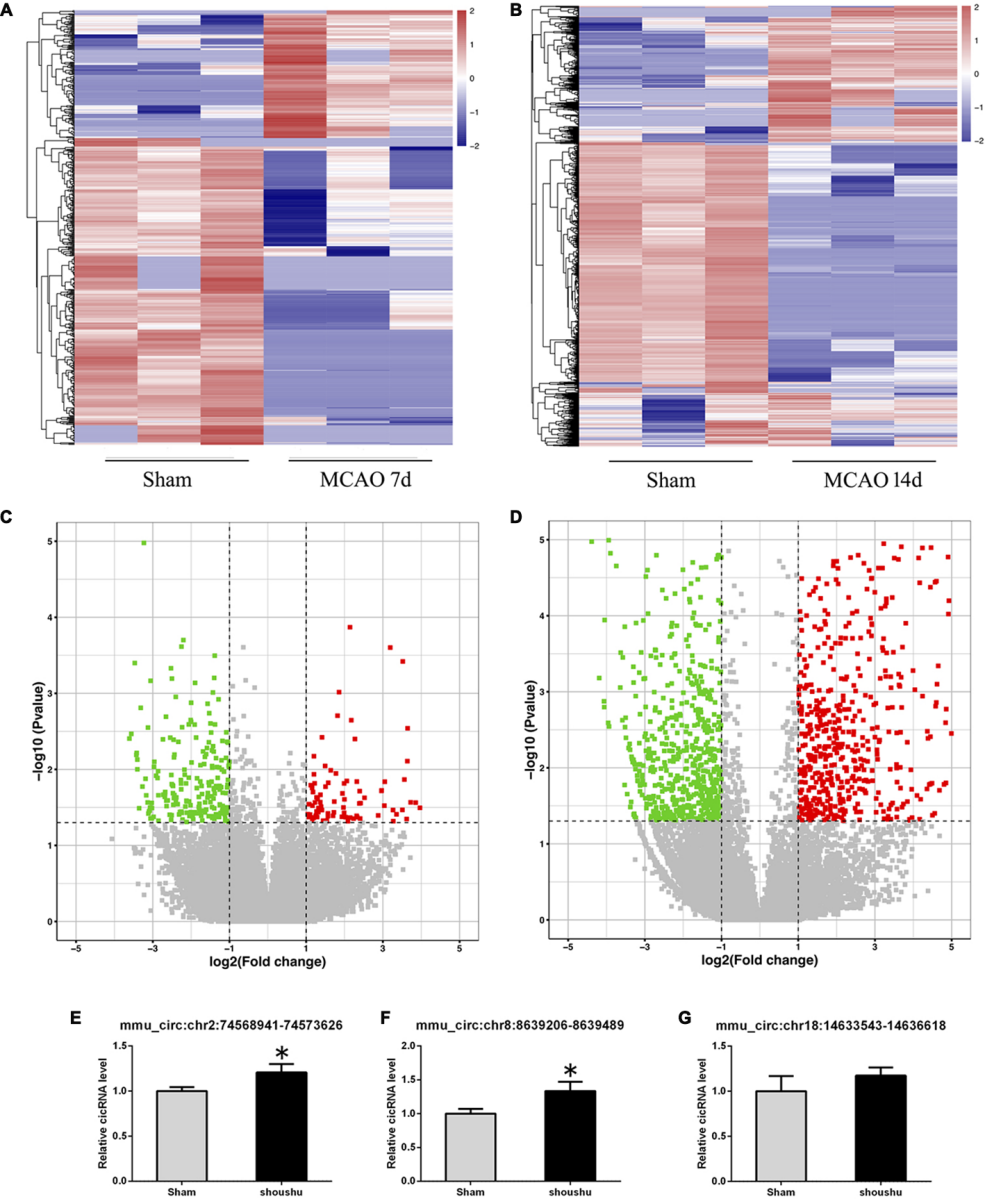
**Altered expression profile of circRNAs in the ipsilateral thalamus after focal cortical infarction in adult mice.** (**A**, **B**) The hierarchical clustering analyses of all differentially expressed circRNAs in the ipsilateral thalamus in the sham and distal middle cerebral artery occlusion (MCAO) groups: Red represents a higher fold change and blue represents a lower fold change. (**C**, **D**) The volcano plots of all the detected circRNAs in both the sham and MCAO groups: The vertical line represents to |log 2 (fold change) | > 1 up and down, respectively, and the horizontal line corresponds to a p-value of 0.05 (−log10 scaled). The red points represent the differentially up-regulated circRNAs and the green point represents the significantly down-regulated circRNAs with statistical significance. (**E**–**G**) The validation results of mmu_circ:chr2:74568941-74573626, mmu_circ:chr8:8639206-8639489, and mmu_circ:chr18:14633543-14636618 by qRT-PCR in the sham and MCAO groups (n=6 each).

**Figure 3:**
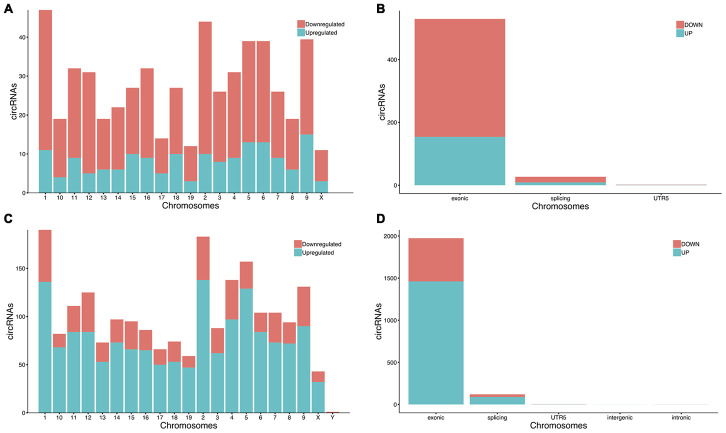
**The chromosomes orientation and gene sources of significantly altered circRNAs in mice.** (**A**, **C**) The significantly altered circRNAs in the mouse ipsilateral thalamus at 7 d and 14 after MCAO were transcribed from all chromosomes. (**B**, **D**) The majority of the differentially expressed circRNAs in the ipsilateral thalamus at 7 d and 14 d after MCAO originated from exonic.

Compared with the sham group, 558 circRNAs (including 164 up-regulated and 394 down-regulated circRNAs) were found to be differentially expressed in the mouse ipsilateral thalamus at 7 d after MCAO, while 2101 circRNAs (including 1475 up-regulated and 626 down-regulated circRNAs) were observed to be significantly altered in the mouse ipsilateral thalamus at 14 d after stroke. The hierarchical clustering heatmaps revealed distinguishable circRNA expression profiles (Figure 2A and 2B), and the volcano plots showed significantly variable expression levels of circRNAs between the sham and MCAO mice (Figure 2C and 2D). The distribution analysis showed that both the up-regulated and down-regulated circRNAs were transcribed from all chromosomes (Figure 3A and 3C). Among all the circRNAs differentially expressed in the ipsilateral thalamus at 7 or 14 d after MCAO, the vast majority were exonic in origin (Figure 3B and 3D).

### Validation of the significantly altered circRNAs in the ipsilateral thalamus after focal cortical infarction

To confirm the accuracy and reliability of the high-throughput results, at 14 d after MCAO, we randomly reselected 3 circRNAs (mmu_circ:chr2:74568941-74573626, mmu_circ:chr8:8639206-8639489, and mmu_circ:chr18:14633543-14636618) that altered significantly at both two points for validation via quantitative real-time polymerase chain reaction (qRT-PCR). The expression level of mmu_circ:chr2:74568941-74573626 and mmu_circ:chr8:8639206-8639489 were found to be up-regulated significantly in the ipsilateral thalamus at 14 d after MCAO compared with the sham mice, which were consistent with the high-throughput results (Figure 2E, 2F). However, the validation result of mmu_circ:chr18:14633543-14636618 was not similar to the high-throughput result (Figure 2G).

### Bioinformatics analyses for the host genes of significantly altered circRNAs

**Figure 4:**
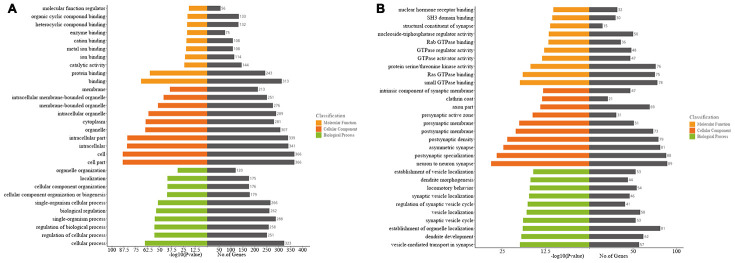
**Gene ontology (GO) analyses of the host genes of significantly altered circRNAs in the mouse ipsilateral thalamus after focal cortical infarction.** (**A**) GO analysis of the host genes of significantly altered circRNAs in the mouse ipsilateral thalamus at 7 d after MCAO. (**B**) GO analysis of the host genes of significantly altered circRNAs in the mouse ipsilateral thalamus at 14 d after MCAO.

To predict the possible biological functions of differentially expressed circRNAs in the mouse ipsilateral thalamus after MCAO, we conducted Gene Ontology (GO) functional analysis on the host genes of significantly altered circRNAs. The top GO terms in the biological processes (BP) were “cellular process, regulation of the cellular process, regulation of the biological, single organism process, and biological regulation” at 7 d after MCAO (Figure 4A), and transformed into “vesicle-mediated transport in synapse, dendrite development, establishment of organelle localization, synaptic vesicle cycle, and vesicle localization” at 14 d after stroke (Figure 4B). The major cellular components (CC) terms were “cell, cell part, intracellular part, organelle, and cytoplasm” at 7 d after MCAO (Figure 4A), and converted into “neuron to neuron synapse, postsynaptic specialization, asymmetric synapse, postsynaptic density, and postsynaptic membrane” at 14 d after stroke (Figure 4B). The most molecular function (MF) terms were “binding, protein binding, catalytic activity, ion binding, and metal ion binding ” at 7 d after MCAO (Figure 4A), and then shifted to following predominance of “small GTPase binding, Ras GTPase binding, protein serine/threonine kinase activity, GTPase activator activity, and GTPase regulator activity” at 14 d after stroke (Figure 4B).

**Figure 5:**
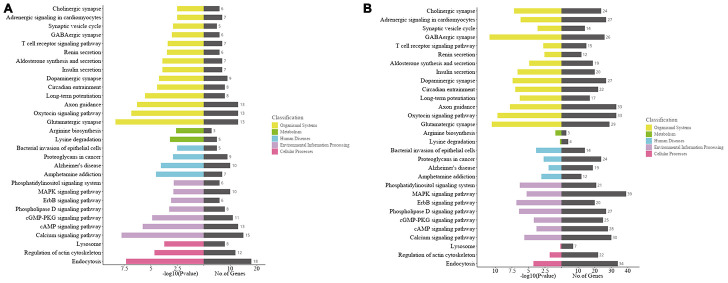
**Kyoto Encyclopedia of Genes and Genomes (KEGG) analyses of the host genes of significantly altered circRNAs in the ipsilateral thalamus after focal cortical infarction in adult mice.** (**A**) KEGG analysis of the host genes of significantly altered circRNAs in the mouse ipsilateral thalamus at 7 d after MCAO. (**B**) KEGG analysis of the host genes of significantly altered circRNAs in the mouse ipsilateral thalamus at 14 d after MCAO.

Gene-enriched Kyoto Encyclopedia of Genes and Genomes (KEGG) pathway analysis of the host genes of significantly altered circRNAs was conduct. The top 30 enriched pathways for the mRNAs transcribed from the host genes were shown in Figure 5B. Among them, “glutamatergic synapse, calcium pathway, oxytocin signaling pathway, endocytosis and axon guidance” were the top 5 signaling pathways of host genes associated with the significantly altered circRNAs in the ipsilateral thalamus at 7 d after MCAO (Figure 5A), while “GABAergic synapse, glutamatergic synapse, oxytocin signaling pathway, axon guidance, and dopaminergic synapse” are the major enriched pathways at 14 d after MCAO (Figure 5B).

### Construction of the circRNA-miRNA-mRNA network and bioinformatics analyses for the target genes of significantly altered circRNAs

**Figure 6:**
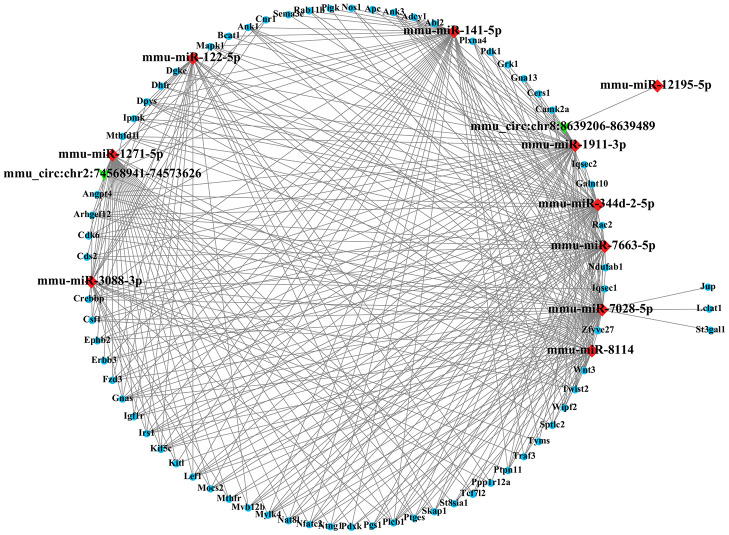
**The circRNA-miRNA-mRNA interaction network.** The network consists of 2 circRNAs, 10 miRNAs and 69 target genes.

**Figure 7:**
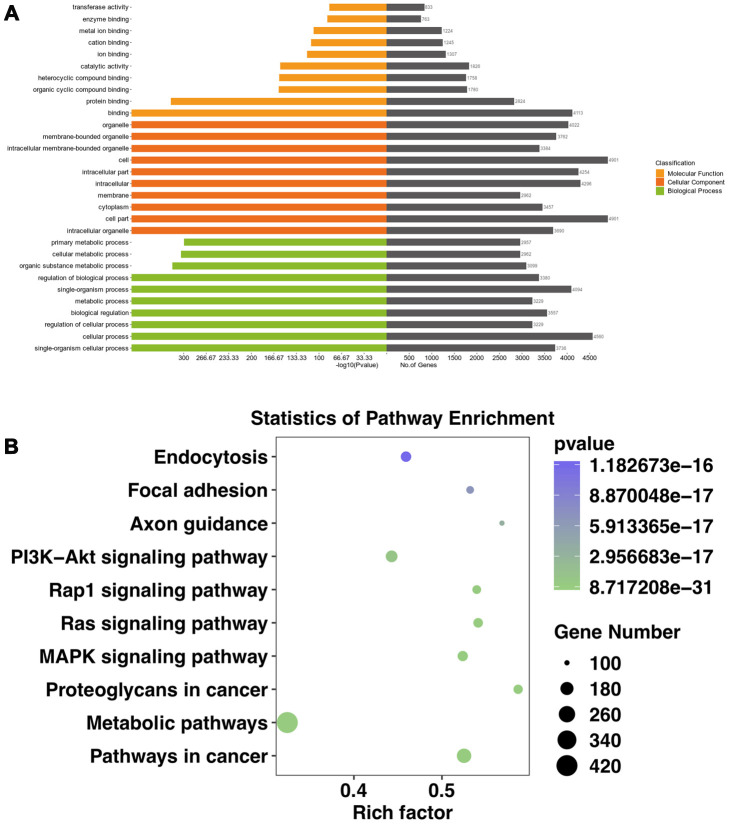
**GO and KEGG analyses of the target genes of significantly altered circRNAs in the mouse ipsilateral thalamus at both two time points (7 and 14 d after MCAO).** (**A**) The GO analysis of the target genes of significantly altered circRNAs. (**B**) The KEGG analysis of the target genes of significantly altered circRNAs.

Most CircRNA can act as miRNAs sponges to remove the inhibition of miRNA on its downstream genes. In this study, we conducted a circRNA-miRNA-mRNA interaction network to predict the competing endogenous RNAs (ceRNA) effect of circRNA which differently expressed in the mouse ipsilateral thalamus after MCAO. The 2 validated circRNAs (mmu_circ:chr2:74568941-74573626 and mmu_circ:chr8:8639206-8639489) were selected and the top 5 representative miRNA response elements for each circRNA were predicted based on the software of Miranda and Targetscan. Next, a total of 1049 target mRNAs were also predicted and obtained via Miranda and Targetscan. With Miranda “total score” >500 as the threshold, 69 genes were screened. Thus, we constructed the circRNA-miRNA-mRNA regulatory network including 2 circRNAs, 10 miRNAs and 69 mRNAs (Figure 6). GO analysis for the target genes of circRNAs showed that the top GO terms in BP were “single organism process, cellular process and regulation of the cellular process”, the major CC terms were “intracellular organelle, cell part, and cytoplasm intracellular part”, and the most MF terms were “binding, protein binding, and organic cyclic compound binding” (Figure 7A). KEGG pathways analysis for the target genes of circRNAs showed that metabolic pathways, pathways in cancer, PI3K-Akt signaling pathway, endocytosis, MAPK signaling pathway, Ras signaling pathway, proteoglycans in cancer, Rap1 signaling pathway, focal adhesion, axon guidance are the top 10 signal pathways associated with the circRNAs altered. These pathways are closely related to inflammatory response and self-repair, which are critical in the non-ischemic remote injury and remodeling after focal cerebral infarction (Figure 7B).

## DISCUSSION

In this study, we found that a total of 2659 circRNAs were differentially expressed in the mouse ipsilateral thalamus after focal cortical infarction. This is the first study to profile the circRNA expression in the non-ischemic remote regions in animal models of ischemic stroke. Bioinformatics analyses predicts that the significantly altered circRNAs may be involved in the pathophysiological processes of secondary thalamic alterations after ischemic stroke.

As early as 1906, von Monakow proposed the concept of diaschisis, which was defined as the functional cessation of brain regions remote from but connected to the primary lesion [18]. In recent years, emerging evidence suggests that focal cerebral infarction not only causes neuronal death in the ischemic infarcts, but also results in secondary neurodegeneration in the non-ischemic remote regions, such as the ipsilateral thalamus, substantia nigra and hippocampus [1–5]. Notably, secondary remote neurodegeneration may impact the long-term outcomes of patients with ischemic stroke. Given that secondary remote neurodegeneration occurs relatively late and progresses slowly after stroke onset, it is more feasible to prevent and treat secondary remote injuries than to treat ischemic injuries.

We used a mouse model of focal cortical infarction in this study. The infarction caused by permanent distal MCAO was only confined to the left cortex, while the ipsilateral thalamus is spared. As we know, the blood supply of thalamus is mainly supplied by the posterior cerebral artery and not affected by MCAO; accordingly, the ipsilateral thalamic damage such as neuronal loss is secondary to the primary cortical infarction, rather than the directly ischemic injury caused by MCAO. Therefore, the mouse with distal MCAO is an ideal animal model to study the secondary thalamic degeneration after focal cortical infarction.

Although the post-stroke secondary changes in the non-ischemic remote regions have attracted more and more attentions, our current understanding of the molecular mechanisms are largely unknown. CircRNA is a special class of endogenous non-coding RNAs with no 5' cap or 3' polyadenylation tail, which is generated during pre-mRNA splicing. CircRNAs are naturally resistant to RNase R and characterized by stable structure, evolutionary conservation and tissue specificity. Accumulated evidences have revealed that circRNAs have essential roles in the regulation of pathophysiological processes of various diseases, such as cancers, autoimmune diseases, cardiac diseases, and neurological diseases [19–24]. CircRNAs are widely expressed in normal brain tissues [9]. Notably, a total of 283 significantly altered circRNAs were found in the ischemic penumbral cortex in mice at 6 and 24 h after cerebral ischemia-reperfusion injury [16]. CircHECTD1 was significantly elevated in the plasma of both acute cerebral infarction mice and patients. Moreover, circHECTD1 promoted autophagy by regulating the miR-142/TIPARP pathway, leading to the loss of neurons and the neurological impairment in mice with cerebral infarction [25]. The aforementioned findings suggest that circRNAs are key regulatory molecules in the pathophysiological processes of the ischemic brain tissues after stroke. However, whether the post-stroke secondary neurodegeneration and remodeling in the non-ischemic remote areas is associated with altered circRNA expressions remains unknown.

The ipsilateral thalamus is one of the most common sites to observe secondary remote neurodegeneration after focal cortical infarction. Previous studies revealed that the main mechanisms of secondary thalamic injury include retrograde and anterograde degeneration of nerve fibers connecting the primary lesion to the ipsilateral thalamus. Inflammation, apoptosis, and oxidative damage were also confirmed in the secondary remote neurodegeneration after ischemic stroke [1, 2, 4, 26–28]. In our previous report, we found newborn blood vessels neuronal cells and in the ipsilateral thalamus of rats with focal cortical infarction, indicating angiogenesis and neurogenesis occurred in the non-ischemic remote region after ischemic stroke [8]. Other studies confirmed that angiogenesis occurred in the rat ipsilateral thalamus following MCAO [29, 30]. In this study, we found 2659 significantly altered circRNAs, including up-regulated and down-regulated circRNAs, in the non-ischemic ipsilateral thalamus at 7 or 14 d after focal cortical infarction in adult mice. Our and others’ previous studies have shown that secondary thalamic changes in the ipsilateral thalamus progress over time after MCAO in animal models [4, 8, 29]. It was interesting that the number of significant altered circRNAs in the ipsilateral thalamus at post-stroke 14 d was much higher than that at 7 d after focal cortical infarction. In addition, our study showed that the differentially expressed circRNAs in the ipsilateral thalamus in mice were all distributed on chromosomes. Most of the differentially altered circRNAs in the ipsilateral thalamus are derived from exons, which is consistent with the findings that exons can produce circRNAs by splicing circulating variables. This is the first study to explore the secondary pathophysiological process in remote areas after ischemic stroke from the perspective of non-coding RNA. According to our results, we suspected that differentially expressed circRNAs may be closely associated with secondary thalamic alterations after focal cerebral infarction in mice.

CircRNAs are produced during the processing of mRNA precursors. It has been reported that some circRNAs play important roles by participating in the regulation of linear transcription of host genes. To better understand the function of significantly altered circRNAs in the mouse ipsilateral thalamus after focal cortical infarction, we conducted GO functional analysis of the mRNAs which are transcribed from the host genes of significantly altered circRNAs. It is well known that the main pathological changes in the ipsilateral thalamus in the early stage of focal cortical infarction are neuronal necrosis and apoptosis, as well as microglia cells activation and proliferation. In this study, we found that the most and meaningful BP and CC terms of circRNAs’ host genes in the ipsilateral thalamus were involved in cellular or intracellular at 7 d after MCAO, and shifted to synapses, synaptic vesicles and dendritic development at 14 d after MCAO, while the MF converted to the activation of enzymes, kinases and formation of synapse at 14 d after MCAO. Our findings revealed that the secondary changes shifted from neural cells damage to self-repair in the non-ischemic thalamus after focal cortical infarction, suggesting that circRNAs may be key regulatory molecules of the pathophysiological evolution of thalamus after MCAO. In the present study, the KEGG pathways analyses for the host genes of significantly altered circRNAs in the ipsilateral thalamus after MCAO suggested that the glutamate synaptic pathway and calcium signaling pathway had the highest correlations with the significantly altered circRNAs. In various pathological states, glutamate is released and accumulated in large quantities in the brain. Excitatory toxicity of glutamate leads to an increase in intracellular Ca2+, which in turn leads to mitochondrial dysfunction, activation of proteases, increase in reactive oxygen species, and release of NO, leading to neuronal death [31–33]. Neuronal death, the major pathological change of the secondary thalamic injury, occurs several days and lasts weeks after stroke onset. Our KEGG analysis indicated that the excitatory toxicity of glutamate and the following increased intracellular Ca2+ may participate in the molecular mechanisms underlying secondary thalamic degeneration following focal cortical infarction. Therefore, understanding of the regulatory mechanisms of the glutamate pathway and calcium signaling pathway would be helpful for developing effective strategies to reduce secondary remote injuries after acute ischemic stroke. We previously demonstrated neurons death, inflammation, angiogenesis, and neurogenesis in the ipsilateral thalamus in MCAO rats [8]. In the present study, our KEGG analyses of the host genes of significantly altered circRNAs showed that other signaling pathways, such as the cAMP signaling pathway, cGMP-PKG signaling pathway, MAPK signaling pathways and axon guidance, were also enriched. The above signaling pathways are known as to be closely associated with inflammation, neural apoptosis, and angiogenesis. Although whether the differentially expressed circRNAs are major regulators in the secondary thalamic changes remains to be investigated, our study provides new insights into the molecule mechanisms underlying secondary thalamic neurodegeneration and remodeling after focal cortical infarction.

Most circRNAs can act as sponges for miRNAs and regulate endogenous gene expressions through competing RNA networks. To predict the ceRNA effect of differently expressed circRNAs in the mouse ipsilateral thalamus after focal cortical infarction, we constructed a circRNA-miRNA-mRNA network in this study. We observed that the mmu_circ:chr2:74568941-74573626 contained sites with both miR-122-5p and miR-1271-5p. It was reported that the expression level of miR-122-5p in plasma and cerebrospinal fluid was significantly down-regulated in rats with transient cerebral ischemia [34]. MiR-122-5p is the upstream modulator of angiogenic factors [35]. MiR-122-5p was also reported be associated with angiogenesis-related biological functions in patients with recurrent ischemic events [36]. MiR-1271-5p inhibited cell proliferation and induces apoptosis by targeting ZIC2 [37]. Besides, miR-1271 significantly inhibited MEK1 and the associated ERK/MAPK signaling pathway [38]. In this study, we found the mu_circ:chr8:8639206-8639489 contained sites with miR-141-5p. It was reported that miR-141-5p reduced cell proliferation, migration, and enhanced cell apoptosis in human leukemic cell line [39]. We speculate that the significantly differentially expressed circRNAs in the non-ischemic thalamus may exert its regulation on downstream target genes through miR-122-5p, miR-1271-5p, and miR-141-5p. Some of the target genes in our circRNA-miRNA-mRNA network, such as EphB2, Wnt3, and Twist1, are mainly involved in inflammation and angiogenesis. It was reported previously that activation of EphrinB2 enhanced angiogenesis and reduced neuronal loss and gliosis in the ipsilateral thalamus in rats with MCAO [40]. The present study revealed the possible mechanism of ephrinB2 in the secondary thalamic neurodegeneration and self-remodeling after cerebral infarction from the perspective of circRNA. In addition, Among the most genes-enrichment pathways of target genes, MAPK, PI3K-Akt, Rap1, and Ras signaling are the downstream cascades of inflammation and angiogenesis, while axon guidance and metabolic pathways are critical in remodeling after the post-stroke secondary remote neurodegeneration. Taken together, our results indicated that the ceRNA effect of circRNAs would be crucial in inflammatory response, neurodegeneration and remodeling in the mouse ipsilateral thalamus after focal cortical infarction. Therefore, the significantly altered circRNAs may be potential targets for reducing secondary neurodegeneration and promoting neural remodeling in non-ischemic regions after ischemic stroke.

There are some limitations in this study. Firstly, we only detected the expression profiles of cortical in the mouse ipsilateral thalamus after focal infarction; the circRNAs expression profiles in other remote regions, such as the substantia nigra and hippocampus, have not been studied. Secondly, we only observed the circRNA expression changes at 7 and 14 d after MCAO, more longer time points should be observed. Thirdly, this is a primary study to profile the circRNA expression in the mouse ipsilateral thalamus after MCAO, further studies are required to investigate the biological function of differentially expressed circRNAs in the non-ischemic remote regions after ischemic stroke.

In conclusion, this is the first study to profile the differentially expressed circRNAs in non-ischemic remote areas after focal cerebral infarction. Bioinformatics analyses predicted that the significantly altered circRNAs are involved in the pathophysiological processes of secondary thalamic alterations after ischemic stroke. Our findings provide new insights into the molecular mechanisms underlying secondary remote degeneration after ischemic stroke, which indicates that circRNAs may be potential therapeutic targets to reduce neurodegeneration and promote neural remodeling after ischemic stroke.

## MATERIALS AND METHODS

### Animals and focal cortical infarction model

Adult male C57BL/6 mice weighing 22~25 g were provided by the Experimental Animal Center of Guangzhou University of Chinese Medicine. The animals were kept in a temperature-controlled room (22 ± 2 °C) under a 12/12 h day/night cycle, with free access to food and water. All animal experiments were approved by the Animal Care and Ethics Committee of Guangzhou University of Chinese Medicine and were performed in accordance with the Guide for the Care and Use of Laboratory Animals published by the National Institutes of Health.

Mico were randomly divided into the focal cortical infarction group and sham-operated group. Focal cortical infarction was induced by permanent distal MCAO. Briefly, mice were anesthetized with isoflurane. Under a surgical microscope, a small burr hole was drilled, and the exposed left distal branch of middle cerebral artery was occluded by bipolar coagulation, followed by 7-min bilateral common carotid occlusion. Animals with symptoms of ischemic stroke were included for further analysis. Mice in the sham-operated group were only exposed the left middle cerebral artery but without occlusion. During the surgical and recovery periods, body temperature was maintained at 37.0 ± 0.5 °C by a heat lamp.

### TTC staining

At 1 d after MCAO, the mice were deeply anesthetized and sacrificed. Their brains were frozen at - 20°C for 5 min, and then 2-mm thick coronal section was made at the level of the thalamus (bregma (-1.58mm~ -2.30mm)), and immediately stained in 2% TTC (Sigma, USA) solution (37°C, 30 min). The red region is normal brain tissue and the white region is infarction lesion.

### H&E staining

At 14 d after MCAO, the paraffin-embedded brain slices were deparaffinized with xylene and gradient alcohol. Next, the slices were stained with hematoxylin for 4 min, washed, and then eosin staining was followed for 3 min. After staining, the slices were dehydrated with ethanol, washed with xylene, and sealed with neutral gums finally.

### Nissl staining

At 14 d after MCAO, fresh brain tissue was fixed in 4% paraformaldehyde with 4°C for 24 h, and then the coronal brain tissue selected from 1mm anterior to 3mm posterior to the anterior fontanelle was routinely dehydrated and embedded with paraffin. Next, 5-μm thick coronal paraffin sections were prepared and then incubated at 60 °C for 2 h. After conventional dewaxing and hydrating, the slices were stained in the reagent of Cresyl violet Stain (37 °C, 30 min), and then rinsed with distilled water for 5min and differentiated with reagent of Nissl differentiation for 1 min. Finally, the slices were dehydrated and sealed with neutral gums.

### RNA extraction and quality control

Mice were deeply anesthetized at 7 and 14 d after the operation, and then perfused transcardiacly with cold (4 °C) isotonic saline. Next, the mouse ipsilateral thalamus at the coronal level of interaural 2.22 mm~1.50 mm and bregma -1.58 mm ~ -2.30 mm was dissected rapidly, and then frozen in liquid nitrogen. Total RNA was extracted using TRIzol reagent (Invitrogen, Carlsbad, CA) according to the manufacturer’s protocol. The purity of total RNA were assessed by a Nanodrop spectrophotometer (ND-1000, NanoDrop Technologies, Wilmington, DE, USA). The integrity of RNA was determined by standard denaturing agarose gel electrophoresis.

### Library preparation and high-throughput sequencing of circRNAs

In brief, ribosomal RNA was removed from total RNA, and then RNase R (Epicentre, USA) were used to digest linear RNAs. Subsequently, purified RNA fragments were subjected to the first strand and second strand cDNA synthesis, and then adapter-ligated and enriched using NEBNext® Ultra™ RNA Library Prep Kit for Illumina (NEB, USA). Finally, DNA was amplified. The quality and concentration of library were measured by the Agilent 2200 TapeStation (Life Technologies, USA). HiSeq 3000 with 2 × 150 bp mode was used for circRNA sequencing. CircRNAs were detected by CIRI2 (Beijing Institutes of Life Science, Beijing, China) and CIRCexplorer2 (Shanghai Institutes for Biological Sciences, Shanghai, China). CircRNA detected by both methods will be considered as an identified circRNA. The junction reads of the predicted circRNAs were corrected by reads per million mapped reads, and then the differential circRNAs were calculated by DESeq method. A |log2FoldChange| of >1 and p-value of < 0.05 were used as thresholds for significantly altered circRNAs.

### qRT-PCR validation

To verify the reliability of the high-throughput sequencing results, three circRNAs ((mmu_circ:chr2:74568941-74573626 (circ-Lnp), mmu_circ:chr8:8639206-8639489 (circ-Efnb2), and mmu_circ:chr18:14633543-14636618 (circ-Ss18)) were selected randomly from those altered significantly at both two points (7 and 14 d after stroke) for qRT-PCR. At 14 d after operation, total RNA was extracted from the ipsilateral thalamus of the focal cortical infarction group and sham group mice (n = 6 each) using TRIzol reagent. Reverse transcription to cDNA was achieved with SuperScript III First-Strand Synthesis System (Invitrogen, USA), and qRT-PCR was conducted with a ViiA 7 Real-Time PCR System (Bio-Rad, USA). Glyceraldehyde 3-phosphate dehydrogenase (GAPDH) was used as the internal control. The sequences of primers of circRNAs and GAPDH were presented in Table 1.

**Table 1:** The sequences of quantitative reverse transcription-PCR primers used for analysis of circular RNAs (circRNAs) and GAPDH.

**circRNA ID**	**gene symbol**	**Primer sequences (5'-3')**
mmu_circ:chr2:74568941-74573626	Lnp	F: CTTGTGGTATCTTCCTGATGAGTTT
		R: GTCCAAAAGAAGTCACAGATCAAAG
mmu_circ:chr8:8639206-8639489	Efnb2	F:CACCATCAAGTTTCAAGAATTCAGC
		R:CCTATCTGTGGGTATAGTACCAGGC
mmu_circ:chr18:14633543-14636618	Ss18	F:TCCAGGTCCTCAGTATCCTAATTAT
		R: GCCATACTGGGAGTTTCCTGGT
GAPDH		F:CACTGAGCAAGAGAGGCCCTAT
		R:GCAGCGAACTTTATTGATGGTATT

### GO and KEGG pathway analyses

GO annotations (http://www.geneontology.org) were performed to explore the function of significantly altered circRNAs’ host or target genes, and KEGG (http://www.genome.jp/kegg/) pathway analyses were conducted to explore the significant pathways associated with significantly altered circRNAs’ host or target genes.

### Annotation for circRNA-miRNA-mRNA interaction network construction

A circRNA-miRNA-mRNA interaction network was conducted based on miRanda (http://www.microrna.org) and TargetScan (http://www.targetscan.org). We selected the 2 validated circRNAs and the top five putative miRNAs of each circRNAs. Miranda and targetscan were also conducted to predict the target genes of miRNAs, and then KEGG analysis was performed to obtain the top 10 gene-enriched pathways of the target genes. With Miranda “total score” >500 as the threshold, a total of 69 genes were screened. Subsequently, we constructed a circRNA-miRNA-mRNA interaction network based on Cytoscape software V3.5.0 (San Diego, USA).

### Statistical analyses

All data, presented as mean ± standard deviation, were analyzed by Statistical Program for Social Sciences (SPSS) 20.0 software (SPSS, Chicago, IL). Student’s t-test was used to determine the statistical significance between two groups. A p-value < 0.05 was considered statistically significant.
